# Spatially Selective Solvation Chemistry by Local Charge Enrichment for Stable Potassium‐Metal Anodes

**DOI:** 10.1002/advs.75327

**Published:** 2026-04-28

**Authors:** Lu‐Kang Zhao, Xi‐Ran Zhao, Zhimin Ding, Yu‐Hua Bian, Xuan‐Chen Wang, Dongdong Zhao, Yizhuo Zhao, Xuan‐Wen Gao, Zhaomeng Liu, Wen‐Bin Luo

**Affiliations:** ^1^ School of Metallurgy and Materials Engineering Liaoning Institute of Science and Technology Benxi Liaoning China; ^2^ Institute for Energy Electrochemistry and Urban Mines Metallurgy School of Metallurgy, Northeastern University Shenyang Liaoning China

**Keywords:** anion‐cation interaction, functional intermediate medium, potassium metal batteries, solvation structures

## Abstract

Uncontrollable dendrite growth and unstable interface chemistry between the electrode and electrolyte seriously hinder the practical application of potassium metal batteries (PMBs). Herein, a functional intermediate medium comprising molybdenum carbide decorated N‐doped carbon (MoC/NC) is designed to preferentially and strongly interact with the KFSI molecules, which causes localized charge rearrangements on its surface and the formation of electron‐rich microdomains. It promotes the local accumulation of K^+^ and induces an anions dominant solvation structure, ultimately achieving the construction of SEI rich in inorganic components such as KF. The derived SEI layer effectively suppresses the electron tunneling effect because of the electronic insulation of KF, while the integrated MoC/NC functional intermediate medium facilitates the rapid and balanced K^+^/electron diffusion due to its intrinsic high ionic conductivity. Benefiting from this interfacial synergy, the integrated electrode (MoC/NC@K) exhibits significantly boosted interfacial transport kinetic and uniform potassium deposition behavior. The symmetric cell demonstrates a stable cycling for more than 800 h at 0.5 mA cm^−2^, and the cycle stability is also enhanced in the full cells. This work presents innovative concepts and approaches for precisely regulating interface components in PMBs by a multi‐scale strategy of molecules and interfaces.

## Graphical abstract

A MoC/NC functional intermediate medium preferentially interacts with KFSI to reconstruct the interfacial solvation structure and form a KF‐rich inorganic SEI. The synergistic integration of the electronically insulating SEI and MoC/NC suppresses electron tunneling, enables fast and balanced K^+^/electron transport, and promotes uniform K deposition for long‐life potassium metal batteries.

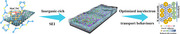

## 1 Introduction

The development of battery systems utilizing earth‐abundant elements is critical to addressing the supply instability of lithium resources [1, 2, 3]. Among these, potassium metal batteries (PMBs) are emerging as a promising next‐generation energy storage technology, driven by the high natural abundance of potassium (2.09 wt.% in the Earth's crust) and its impressive theoretical specific capacity of 687 mAh g^−1^ [4, 5]. However, the practical deployment of PMBs is severely hindered by the uncontrolled growth of potassium dendrites and the inherent instability of the solid electrolyte interphase (SEI) [6, 7, 8].

**FIGURE 1:**
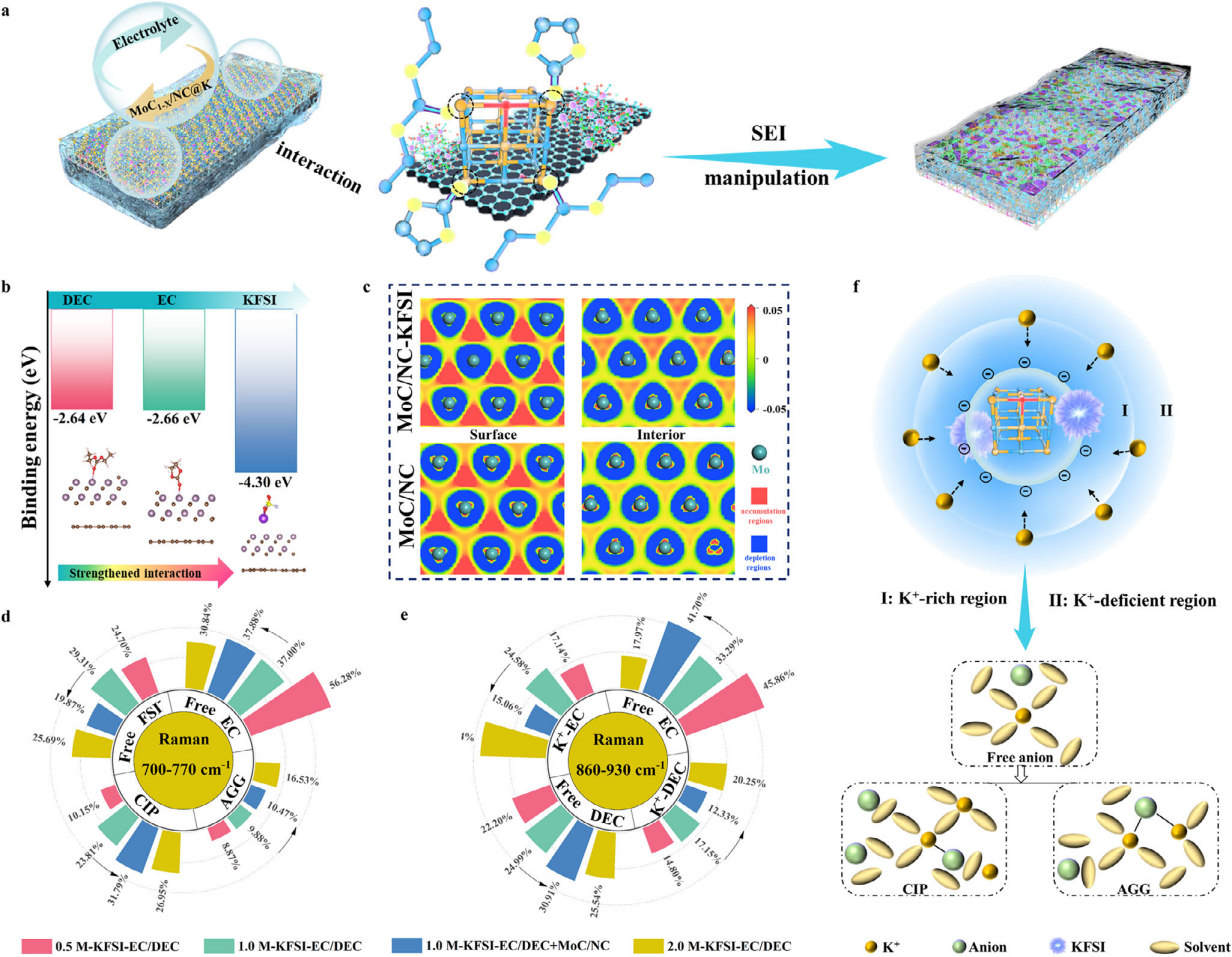
(a) Schematic illustration of the interaction between the functional MoC/NC and the electrolyte. (b) Adsorption energy of key molecules in the electrolyte by MoC/NC. (c) The cross‐sectional color‐filled diagrams of differential charge density of MoC/NC before and after adsorbing KFSI molecules. Proportions of solvation structures identified by Raman spectra in the electrolyte of 0.5 m KFSI‐EC/DEC, 1.0 m KFSI‐EC/DEC, 1.0 m KFSI‐EC/DEC with MoC/NC, and 2.0 m KFSI‐EC/DEC in the range of (d)700‐770 cm^−1^ and (e) 860–930 cm^−1^. (f) Diagram illustrating the influence of MoC/NC particles on the solvation structure of ions.

Conventional strategies, such as 3D collectors or inorganic‐rich artificial SEI formed via concentrated electrolytes and additives, partially address these issues but often suffer from side reactions, high viscosity, or poor interfacial regulation [9, 10, 11, 12, 13, 14]. Recently, constructing an integrated electrode by mechanically embedding a functional intermediate medium (e.g., via repeated cold rolling and folding) has emerged as a transformative solution to homogenize ion flux and alleviate internal stress [15, 16, 17]. Despite these mechanical benefits, the potential of the intermediate medium remains underutilized. Most existing studies focus on physical confinement or ion transport regulation, largely overlooking the active modulation of the nanoscale solvation structure within the intermediate medium to electrolyte. Given that the primary solvation sheath dictates the SEI composition, engineering a functional intermediate medium capable of actively regulating ion–solvent interactions is pivotal for achieving stable potassium metal anode (Figure 1a) [18, 19].

Embedding electronically active sites that preferentially interact with anions such as FSI^−^ can weaken cation‐solvent coordination and reshape the interfacial solvation environment, thereby facilitating the formation of an inorganic‐rich SEI. Transition metal carbides (TMCs) exhibit tunable electronic structures and strong anion‐binding tendencies. Among them, molybdenum carbide (MoC) is particularly attractive because its surface states are conducive to interactions with the F and S atoms in FSI^−^, enabling effective anion adsorption and solvation modulation. Inspired by these features, we herein design a MoC‐decorated N‐doped carbon framework (MoC/NC) and integrate it into the potassium metal anode. Owing to its strong affinity toward KFSI, MoC/NC induces local K^+^ enrichment and promotes weakly solvated structures dominated by contact ion pairs (CIPs) and aggregates (AGGs). This drives preferential FSI^−^ reduction and in situ formation of a robust KF‐rich SEI. The KF framework enhances mechanical integrity and suppresses dendrites, while the potassiophilic and ionic conductivity of MoC/NC ensure the efficient K^+^ transport and uniform deposition. As a result, MoC/NC@K electrodes deliver stable interfaces and superior electrochemical performance in both symmetric and full cells.

## 2 Results and Discussion

The as‐synthesized MoC/NC was characterized by XRD, showing diffraction peaks at 36.9°, 42.0°, 61.6°, and 73.8°, corresponding to the (111), (200), (220), and (311) planes (Figure S1a) [20, 21]. Raman spectroscopy reveals a characteristic D band at 1327.8 cm^−1^ and G band at 1604.9 cm^−1^. The calculated I_D_/I_G_ ratio of 1.37 indicates a defect‐rich carbon framework resulting from nitrogen incorporation [22, 23], which is essential for maximizing the exposure of active MoC sites and enhancing electrochemical reactivity (Figure S1b). Morphologically, the MoC/NC is composed of densely interwoven nanosheets that assemble into a porous 3D architecture (Figure S2). This interconnected porous structure is critical for its role as an intermediate medium, as it facilitates rapid electrolyte infiltration while providing a robust scaffold to buffer the volume fluctuations during potassium plating and stripping [24, 25, 26]. Furthermore, XPS confirms the coexistence of Mo^2+^, Mo^4+^, and Mo^6+^ (228.6, 229.5, and 233.1 eV, implying abundant redox‐active sites and possible electron‐trapping effects (Figure S3) [27]. The presence of these multivalent states suggests abundant redox‐active sites and implies potential for electron‐trapping effects that mitigate undesired side reactions. The C *1s* spectra display C─Mo (284.2 eV), C─C (284.8 eV), and C─N (286.1 eV) bonds [28], while O 1s reveals O─C (530.6 eV), O─N (531.3 eV), and C─O─C (533.0 eV) [29]. Meanwhile, the N *1s* high resolution spectra shows a mixture of Mo─N (395.1 eV), pyrrolic N (399.1 eV) [28, 30], and Mo 3p/Mo^4+^ contributions at 394.2 and 397.4 eV [31]. These results verify the successful incorporation of N‐doped carbon into the MoC matrix, which not only improves electronic conductivity but also modulates interfacial electron distribution.

DFT calculations were performed to assess the interaction affinity of the MoC/NC toward solvent and salt species. As shown in Figure 1b and Figure S4, MoC/NC shows stronger adsorption toward KFSI (−4.30 eV) than that of EC (−2.66 eV) and DEC (−2.64 eV), suggesting it preferentially facilitates the reductive decomposition of KFSI, while suppressing premature solvent breakdown. To clarify the interfacial effects of KFSI adsorption, differential charge density mapping was performed. As shown in Figure 1c, significant charge redistribution occurs in both the interfacial and bulk regions of MoC/NC upon KFSI adsorption, particularly around Mo atoms. In detail, the significant polarization is induced by the charge redistribution, which results in the formation of electron‐rich domains at the interface. The formation of electron‐rich domains at the interface provides anchoring sites for dissociated K^+^ from KFSI via electrostatic attraction, resulting in the establishment of locally high concentrated K^+^ regions. The interaction between dissociated K^+^ and FSI^−^ anions was further examined by calculating the Fukui functions of both KFSI and isolated FSI^−^ anions (Table S1). Notably, in this analysis, only the nucleophilic properties of individual atoms within the FSI^−^ anions and their counterparts in KFSI were considered, while the nucleophilicity of the K^+^ itself was excluded. Compared to the molecular KFSI, the FSI^−^ anions demonstrates a consistently enhanced nucleophilic character across all relevant atomic sites. Such enhancement suggests that, once K^+^ ions accumulate at the MoC/NC‐electrolyte interface and generate electropositive domains, free FSI^−^ anions are more susceptible to nucleophilic attraction. The enhanced probability of such interactions promotes the formation of CIPs and AGGs in the electrolyte. FSI^−^ anions coordinated with K^+^ can thus be efficiently incorporated into the electric double layer and participate in interfacial electrochemical reactions, leading to the generation of FSI^−^ anions‐derived inorganic SEI components such as KF on the MoC/NC surface [32]. Such a mechanism effectively mitigates excessive decomposition of solvents and solutes, contributing to the construction of a more stable and robust SEI.

To investigate the effect of MoC/NC on the solvation structure of K^+^ in the electrolyte, Raman spectroscopy was conducted on KFSI‐EC/DEC systems with different salt concentrations and with/without MoC/NC (Figure 1d,e; Figure S5). In the range of 700–770 cm^−1^, a clear decrease in the proportion of free EC is observed with increasing salt concentration. Specifically, the free EC content decreases from 56.3% in 0.5 m KFSI to 37.0% in 1.0 m and then to 30.8% in 2.0 m. Simultaneously, the content of CIP and AGG increases, indicating enhanced coordination between K^+^ and FSI^−^ and reduced interaction with solvent molecules. Similar trends are observed in the 860–930 cm^−1^ region, where the coordination peaks of K^+^‐EC and K^+^‐DEC appear. In 1.0 m, the EC and DEC coordination ratios reach 24.6% and 17.1%, respectively, confirming that higher salt concentration promotes K^+^‐anion coordination while suppressing solvent involvement. Upon introducing MoC/NC into the baseline 1.0 m KFSI‐EC/DEC electrolyte, the solvation environment changes significantly (Tables S2 and S3). The proportion of K^+^‐EC and K^+^‐DEC coordination decreases markedly, while the content of CIPs and AGGs increases to 31.8% and 10.5%, respectively. Notably, the proportion of free solvent molecules increases compared to the baseline 1.0 m KFSI‐EC/DEC electrolyte, suggesting that MoC/NC promotes the preferential coordination of K^+^ with FSI^−^, thereby weakening its interaction with solvent molecules. This is attributed to the surface sites of MoC that can enhance FSI^−^ enrichment around K^+^, facilitating the formation of an anion‐dominated solvation sheath. Furthermore, the total (CIP + AGG) ratio in the MoC/NC‐modified 1.0 m electrolyte reaches 42.3%, approaching the value of the 2.0 m high‐concentration system (43.5%), despite using a lower salt concentration. Meanwhile, the lower K^+^‐solvent coordination ratio highlights the efficient suppression of solvent participation in K^+^ solvation. As summarized schematically in Figure 1f, the synergistic effect of MoC/NC and FSI^−^ anions establishes a dual‐region solvation environment, where Region I represents a K^+^‐rich zone dominated by strong K^+^‐FSI^−^ coordination near MoC/NC, while Region II corresponds to a K^+^‐deficient area characterized by weakened solvent interaction. This anion‐rich solvation structure is conducive to the formation of a robust, inorganic‐rich SEI layer, consistent with the DFT calculation results [32, 33, 34].

Based on these favorable attributes, the integrated electrode (denoted as MoC/NC@K) was fabricated by mechanically embedding the MoC/NC intermediate medium into the K metal via a repeated cold‐rolling and folding process (Figures S6 and S7). This technique ensures the structural continuity, yielding an integrated electrode characterized by a continuous morphology and uniformly distributed Mo and C, as confirmed by elemental mapping (Figure S8).

**FIGURE 2:**
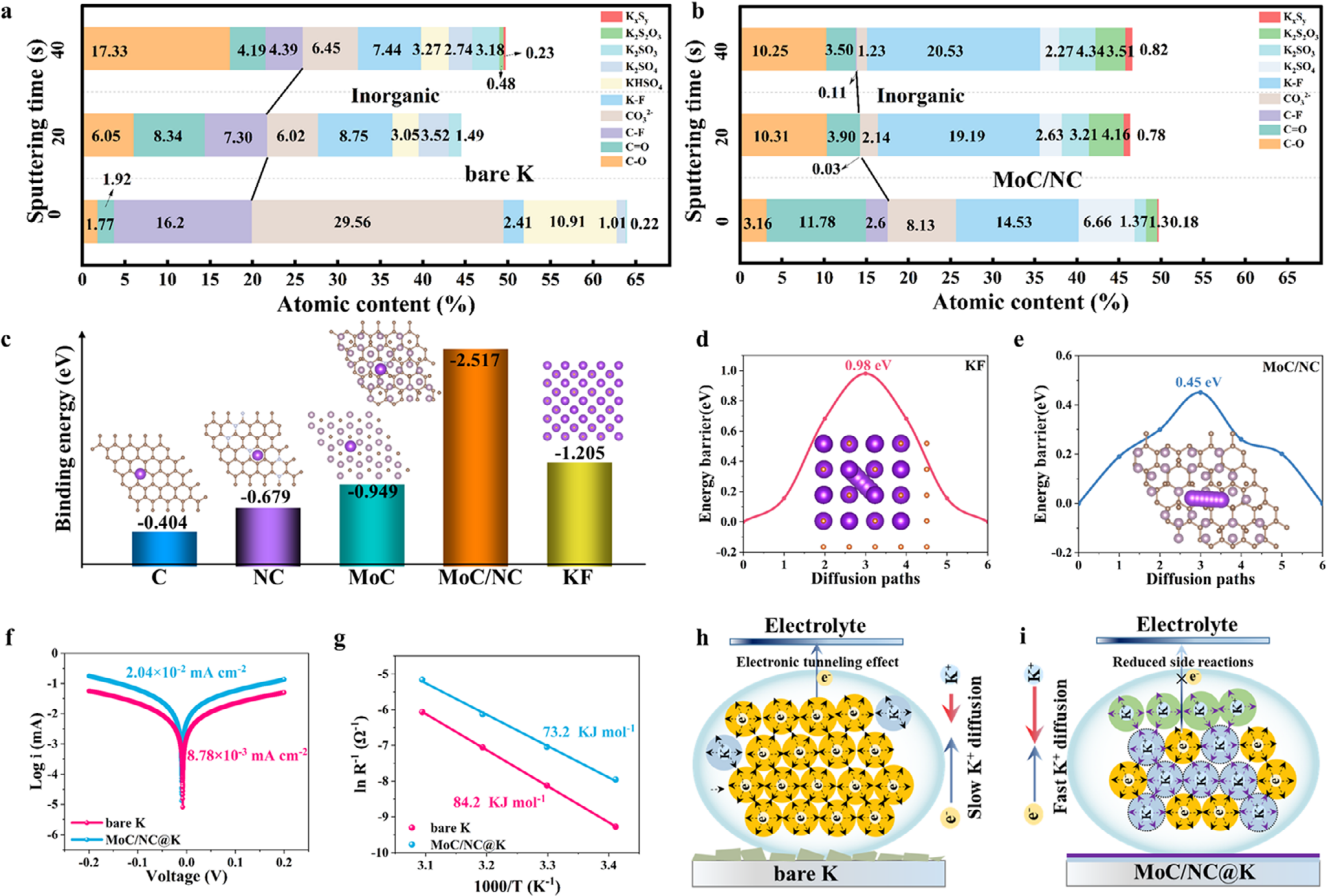
Analysis of surface chemical bonds of (a) bare K and (b) MoC/NC@K after cycling. (c) The adsorption of K^+^ in C, NC, MoC, MoC/NC, and KF. The diffusion of K^+^ in (d) KF and (e) MoC/NC. (f) The Tafel plots of the MoC/NC@K and bare K. (g) The fitted desolvation energy barrier of MoC/NC@K and bare K. The schematic diagrams of the impact of the SEI on electronic and ionic transport for (h) bare K and (j) MoC/NC@K electrodes.

To elucidate the composition of the SEI derived from the modified solvation structure and its impact on ion transport behavior, depth‐profile XPS was performed on symmetric cells after cycling. The cells were disassembled, and the electrodes were subjected to Ar^+^ sputtering for various durations. As shown in Figure S9a,d, two peaks located at 682.7 and 683.5 eV can be assigned to KF species [35]. Throughout the sputtering process, the MoC/NC@K exhibits a notably higher KF content compared to the bare K. This can be attributed to the decomposition of (AGG + CIP) solvation structures in the MoC/NC@K, which leads to the formation of anions‐ derived SEI throughout the depth direction. The peak around 688 eV can be attributed to C‐F species, which are generally considered by‐products of SEI destruction and repeated reconstruction processes [36]. The surface of bare K shows a significantly higher content of C‐F than that of the MoC/NC@K, indicating bare K possesses a less stable SEI and is prone to continuous breakdown and reformation. In contrast, only trace content of C‐F are detected on the surface of the MoC/NC@K, and the signal diminishes to nearly undetectable levels with increased sputtering time (e.g., at 20 and 40 s). This contrast in both the intensity and distribution of C‐F species suggests that the SEI formed on MoC/NC@K is more stable than that formed on bare K. Furthermore, in the S *2p* high resolution spectra, a distinct K_x_S_y_ signal is detected in the MoC/NC@K and becomes increasingly prominent with deeper sputtering, whereas only a faint K_x_S_y_ signal is observed in the K metal electrode at 40 s (Figure S9b,e). The presence of K_x_S_y_, widely recognized as an ion‐conductive species [37, 38], suggests facilitated K^+^ transport through the SEI layer in MoC/NC@K. Based on the elemental compositions and chemical bond proportions at different sputtering depths, the inorganic and organic components within the SEI of both electrodes were quantitatively compared (Figure 2a,b; Figure S10). At the outermost surface of the SEI, the bare K exhibits a higher proportion of K_2_CO_3_ compared to the MoC/NC@K (Figure 2a,b; Figure S9c,f). This carbonate species is typically associated with severe SEI degradation, repetitive reconstruction, and extensive parasitic reactions occurring at the K metal surface [39]. In contrast, compared to that on the bare K, the SEI on the MoC/NC@K contains a greater amount of beneficial inorganic species, such as KF, K_2_SO_4_, K_2_SO_3_, K_2_S_2_O_3_, and K_x_S_y_ [6, 40]. Among these, inorganic compounds such as KF play a crucial role in enhancing the mechanical stability of the SEI, thereby improving its resistance to dendrite penetration while reducing electrolyte consumption and mitigating active potassium loss caused by continuous SEI breakdown and reformation [35, 38]. Additionally, K_x_S_y_ species are widely recognized as efficient ion conductors that enable rapid K^+^ transport through the SEI. This ensures the prompt replenishment of depleted K^+^ near the anode surface, prevents the formation of space‐charge regions, and consequently suppresses dendritic growth effectively.

DFT calculations were performed to further understand the K^+^ transport behavior within the SEI. As shown in Figure 2c, to better understand the role of MoC/NC, its individual building blocks C, NC, and MoC were first examined. Notably, the K^+^ adsorption energy on NC (−0.679 eV) is stronger than that on pristine C (−0.404 eV), indicating that the nitrogen functionalities do contribute to K^+^ adsorption by increasing the local polarity/defect density of the carbon framework. Therefore, the role of N species should not be considered strictly limited to conductivity enhancement. However, this contribution is still secondary relative to the MoC related sites, since MoC shows a stronger K^+^ adsorption energy (−0.949 eV), and the integrated MoC/NC framework exhibits the strongest interaction (−2.517 eV). In addition, the differential charge density analysis upon KFSI adsorption shows the most pronounced charge redistribution around the Mo‐related regions, suggesting that the MoC sites play the dominant role in attracting FSI^−^ and regulating the interfacial solvation structure. Therefore, the nitrogen functional groups provide a synergistic contribution by improving the potassiophilicity and tuning the local electronic environment of the carbon framework, whereas the MoC active sites remain the principal centers responsible for K^+^ enrichment and FSI^−^ involved solvation restructuring. Beyond favorable strong adsorption, MoC/NC presents a low K^+^ diffusion energy barrier of 0.45 eV (Figure 2d), suggesting its potential as a fast ion‐conductive phase in the SEI. In comparison, KF demonstrates a moderately strong K^+^ adsorption energy of −1.205 eV, yet suffers from a higher diffusion barrier of 0.98 eV (Figure 2e), which could limit its contribution to ionic conductivity. Nevertheless, KF plays a vital role in ensuring the structural and electrochemical integrity of the SEI. Its wide bandgap provides a high electronic tunneling barrier, effectively suppressing electron leakage and side reactions at the interface between electrode and electrolyte. These results reveal a cooperative interaction between MoC/NC and KF. While KF contributes to SEI stability and electronic insulation, MoC/NC provides rapid ion transport pathways that compensate for the diffusion limitations of KF. This interfacial synergy ensures efficient K^+^ migration across the SEI and contributes to uniform deposition and enhanced stability of the K metal anode.

During the deposition process of K^+^, it must overcome a nucleation energy barrier to acquire electrons and form metallic K nuclei. To investigate the influence of MoC/NC on K^+^ nucleation behavior, half‐cells of K||Cu and MoC/NC@Cu||K were assembled. As shown in Figure S11, K^+^ nucleation overpotential on bare Cu is 166.2 mV, which is significantly higher than the 9.9 mV observed for MoC/NC@Cu. This pronounced difference confirms that the introduction of MoC/NC effectively reduces the energy barrier for K^+^ nucleation, promoting more uniform and controlled deposition [41]. To further validate the effect of the MoC/NC and its derived SEI layer on interfacial kinetics, Tafel analysis was conducted. As shown in Figure 2f, the MoC/NC@K exhibits a notably higher exchange current density (2.04 × 10^−2^ mA cm^−2^) than the bare K (8.78 × 10^−3^ mA cm^−2^), suggesting improved charge transfer kinetics and accelerated K^+^ transport across the SEI layer in MoC/NC@K [42, 43]. To explore desolvation energy barrier of two electrode, electrochemical impedance spectroscopy were collected under various temperatures (Figure 2g; Figure S12). Based on Arrhenius equations, the calculated desolvation energy barriers are 84.2 kJ mol^−1^ for the bare K and 73.2 kJ mol^−1^ for MoC/NC@K. The lower activation energy in the MoC/NC@K indicates a more favorable desolvation process, which facilitates the faster interfacial reactions [44, 45]. Complementary results from galvanostatic intermittent titration technique (GITT) tests further support these results. As shown in Figure S13, the MoC/NC@K demonstrates reduced polarization voltage and enhanced diffusion behavior during cycling, reflecting more efficient K^+^ transport. Enhanced ionic conductivity at the interface effectively mitigates concentration polarization caused by local ion depletion near the K metal surface, which extends the Sand time and plays a critical role in suppressing dendritic growth during repeated cycling [15, 46]. According to the above results, as illustrated in Figure 2h,i, MoC/NC serves as a functional intermediate medium that promotes the formation of a KF‐rich inorganic SEI layer, which effectively suppresses side reactions caused by electron leakage into the electrolyte and enhances interfacial mechanical stability to inhibit dendrite growth. Furthermore, the superior ionic transport properties of MoC/NC compensate for the relatively limited ionic conductivity of KF, ensuring a balanced and synergistic relationship between ionic and electronic transport rates, thereby achieving fast and uniform K^+^ deposition. In contrast, the bare K anode exhibits limited regulatory effects on the electrolyte, resulting in the formation of an SEI layer with poor ionic conductivity and high organic content. This leads to inhomogeneous and sluggish K^+^ flux distribution, ultimately triggering irregular and severe dendrite growth.

**FIGURE 3:**
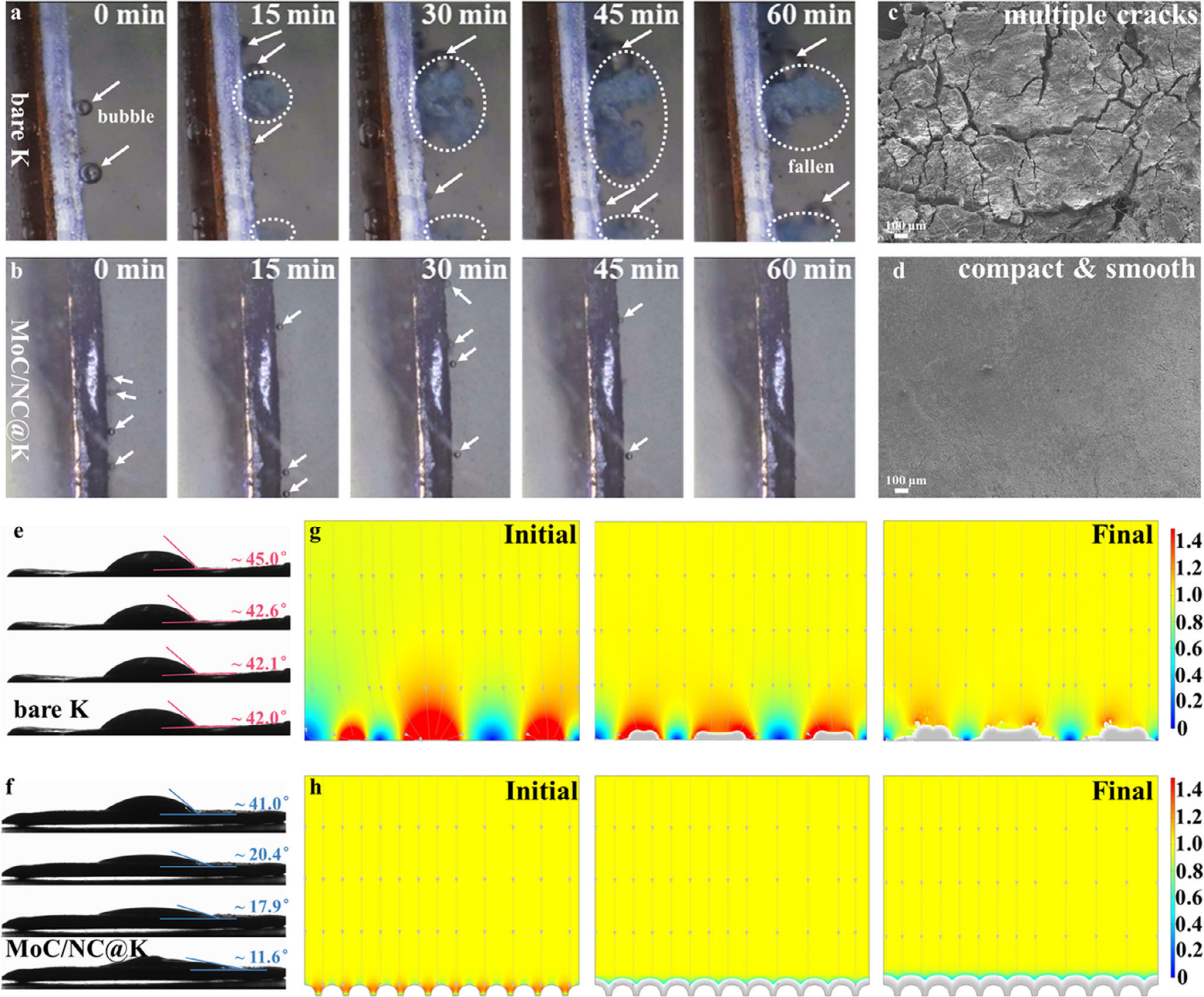
In situ optical observation during the deposition process of the (a) bare K and (b) MoC/NC@K. SEM images of the (c) bare K and (d) MoC/NC@K after long‐term cycling. The wettability of the electrolyte on the (e) bare K and (f) MoC/NC@K. The simulated current density distribution during the deposition process of the (g) bare K and (h) MoC/NC@K.

The K^+^ deposition behavior was further investigated via in situ optical microscopy. As shown in Figure 3a,b, the bare K exhibits obvious dendritic growth within merely 15 min, where the loosely packed spherical aggregates rapidly expand over time. Between 45 and 60 min, the non‐uniformly deposited K structures started to detach from the current collector and eventually fell off, resulting in electrically isolated dead K. In contrast, MoC/NC exhibits a uniform and dendrite‐free deposition surface even after 60 min. This can be attributed to its porous structure, which effectively accommodates volume fluctuations during the deposition process. Furthermore, the uniformly distributed potassiophilic MoC/NC intermediate medium significantly reduces the nucleation barrier, guiding a planar and homogeneous K^+^ deposition process. The long‐term morphological evolution of the electrodes after repeated stripping/plating cycles was examined via SEM and EDS. The bare K surface exhibits disordered dendritic growth along with severe cracking and void formation, primarily attributed to unstable SEI and limited ion transport (Figure 3c; Figures S14a and S15). In contrast, MoC/NC@K retains a smooth, dendrite‐free surface after long‐term cycling, with elemental mapping verifying a uniform distribution of key elements (Figure 3d; Figures S14b and S16), highlighting the structural and interfacial advantages of the MoC/NC architecture in enabling uniform K^+^ deposition and efficient ion transport. The interfacial wettability of 1.0 m KFSI in EC/DEC on bare K and MoC/NC@K was investigated by dynamic contact angle analysis (Figure 3e,f). The MoC/NC@K surface exhibits markedly improved electrolyte affinity, as evidenced by a rapid reduction in contact angle from ∼41° to ∼11.6° within 5 s. In contrast, the bare K maintains a relatively constant angle of ∼42°, indicating the limited wettability. Enhanced electrolyte wettability promotes uniform ion distribution at the interface, mitigates K^+^ concentration polarization, and enables homogeneous ionic flux across the MoC/NC@K surface, thereby contributing to stabilized deposition and effective dendrite suppression. To further elucidate the distinct deposition morphologies, finite element simulations were conducted to analyze the transient current density distribution (Figure 3g,h). For the bare K, the sparse distribution of nucleation sites restricts metal growth to limited regions. As the current density increases, localized deposition becomes more pronounced, promoting the formation of dendritic structures and inducing significant heterogeneity in the interfacial electric field, along with notable field distortion. In contrast, MoC/NC@K exhibits uniformly distributed nucleation sites with strong affinity to K, enabling well‐balanced interactions among ionic flux, electron transport, and active nucleation centers. Such a configuration promotes homogeneous current distribution and suppresses local current accumulation. Consequently, while the bare K displays severe dendritic growth, the MoC/NC@K maintains a more uniform electric field and ion flux, resulting in a smooth and compact deposition morphology.

**FIGURE 4:**
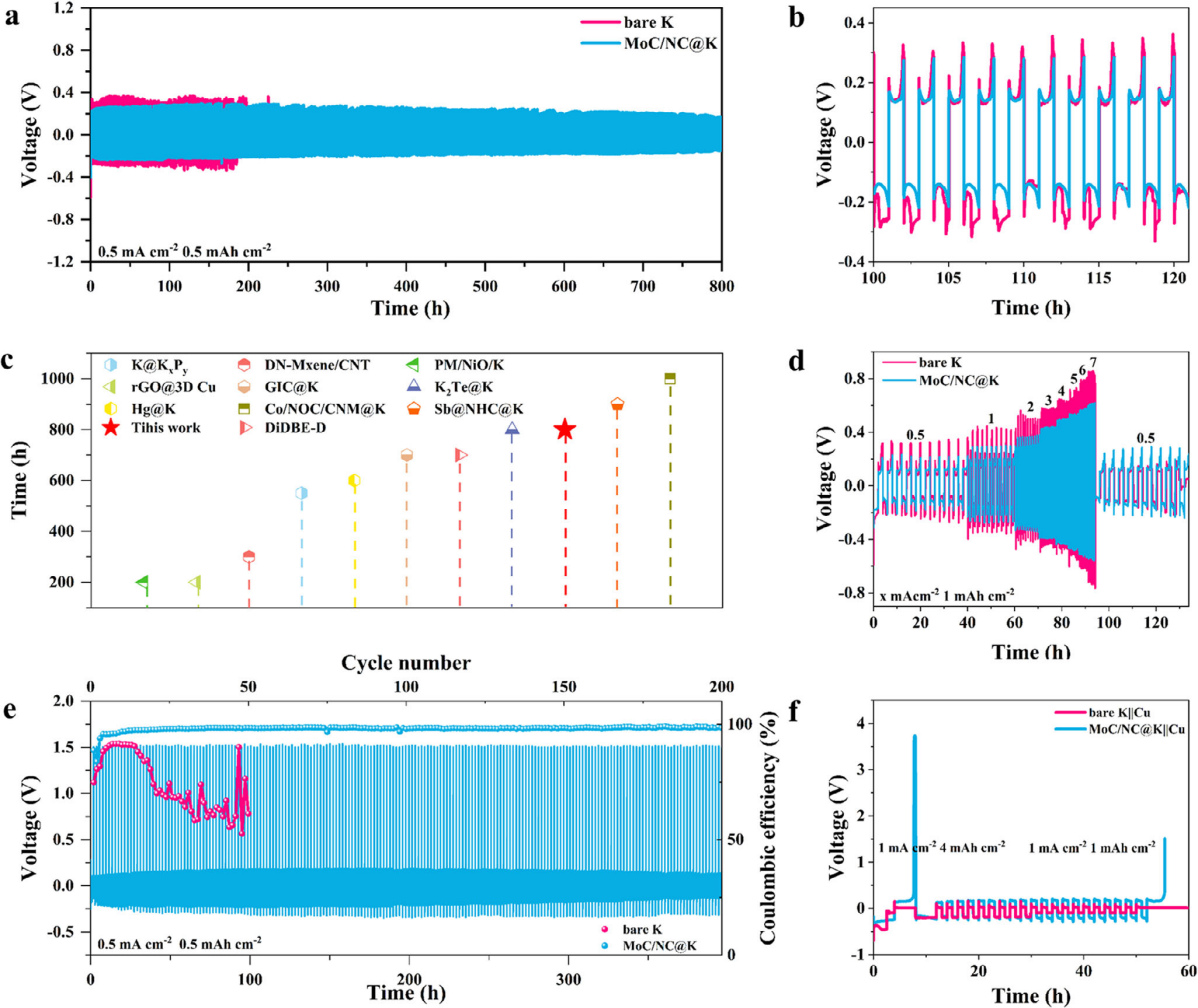
(a) Cycling stability and (b) their enlarged time‐voltage curve of the bare K and MoC/NC@K symmetric cells. (c) Comparison of the cycling performance of symmetric cells with the reported literature. (d) Rate performance of the bare K and MoC/NC@K symmetric cells. (e) The coulombic efficiency of the bare K||Cu and MoC/NC@K||Cu. (f) The average Coulombic efficiency of bare K and MoC/NC@K.

To evaluate the reversibility of different electrodes during long‐term deposition and stripping, symmetric cells were assembled. Under a current density of 0.5 mA cm^−2^ and a plating/stripping capacity of 0.5 mAh cm^−2^, the MoC/NC@K maintains a low polarization voltage of approximately 170 mV after 800 h of cycling (Figure 4a). In contrast, the bare K exhibits a short circuit after around 200 h due to severe dendrite growth and separator penetration. The MoC/NC@K shows a stable voltage‐time profile throughout cycling, indicating significantly improved interfacial stability compared to the bare K (Figure 4b). When compared with a range of previously reported advanced electrode systems in carbonate‐based electrolytes, MoC/NC@K demonstrated superior electrochemical performance (Figure 4c) [10, 15, 17, 26, 41, 46, 47, 48, 49, 50]. Rate capability was further assessed by fixing the deposition capacity at 1 mAh cm^−2^ and varying the current density from 0.5 to 7 mA cm^−2^ (Figure 4d). Across this range, the MoC/NC@K consistently exhibits lower polarization. Even at 7 mA cm^−2^, the voltage hysteresis remains around 620 mV, substantially lower than the 850 mV recorded for the bare K. Coulombic efficiency is further analyzed in half cells under 0.5 mA cm^−2^ and 0.5 mAh cm^−2^. MoC/NC@K sustains an average efficiency of 98.06% for over 200 cycles (Figure 4e), while bare K barely reaches 90% in the early cycles and deteriorates rapidly after the 15th cycle. At a higher current density of 1 mA cm^−2^, MoC/NC continues to deliver 97.55% average efficiency over 20 cycles (Figure 4f). In contrast, the bare K suffers from severe loss of active K, leading to soft and eventually hard short circuits. The improved performance of MoC/NC@K is attributed to its ability to regulate the local solvation environment, promote the formation of an inorganic‐rich SEI, reduce the nucleation barrier, and homogenize the electric field. These combined effects suppress dendritic growth, facilitate uniform K deposition, and ensure excellent interfacial reversibility and long‐term cycling stability.

**FIGURE 5:**
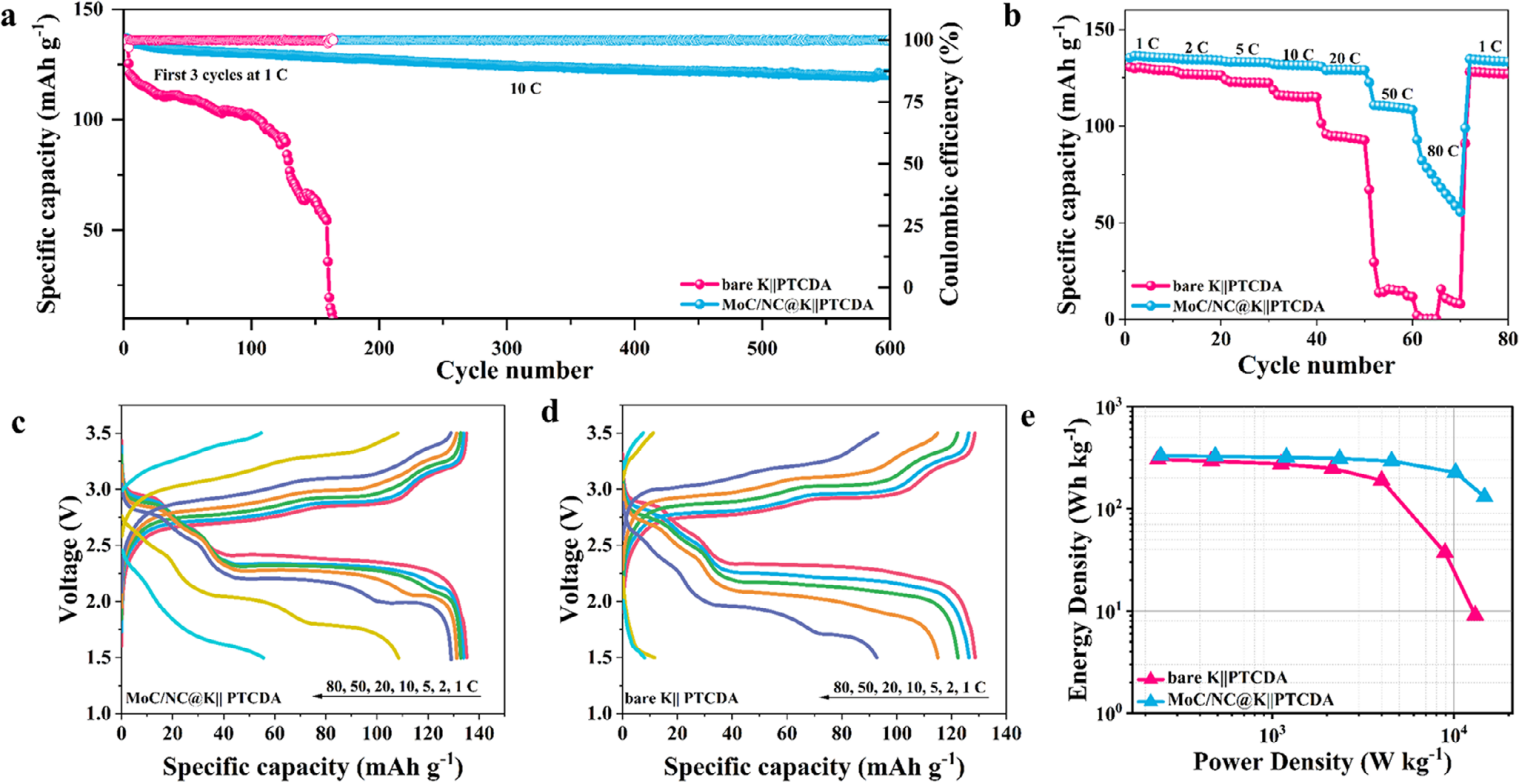
(a) The long cycling performance and (b) rate performance of bare K and MoC/NC@K full‐cells. Charge and discharge curves of (c) MoC/NC@K and (d) bare K full‐cells. (e) Ragon plots of bare K and MoC/NC@K full‐cells.

The full cells employing PTCDA as the cathode were assembled to further validate the interfacial performance (Figures S17 and S18). After 600 cycles at 10  C (1 C = 100 mA g^−1^), the MoC/NC@K||PTCDA cell retains 89.1% of its capacity, delivering 120.0 mAh g^−1^ (Figure 5a). In comparison, the K||PTCDA cell exhibits a sharp decline to below 80% within 105 cycles and drops below 10.0 mAh g^−1^ thereafter, reflecting severe interfacial degradation. The rate capability is systematically evaluated across a wide range of 1 C to 80 C, as shown in Figure 5b. Even at 80 C, MoC/NC@K||PTCDA delivers 55.6 mAh g^−1^, whereas bare K||PTCDA exhibits negligible capacity output. In addition, the voltage profiles further confirm the reduced polarization and improved interfacial kinetics provided by the MoC/NC intermediate medium (Figure 5c‐d). The corresponding Ragone plots indicate an energy density of 318.6 Wh kg^−1^ at 1196.1 W kg^−1^, while 130.6 Wh kg^−1^ is still maintained at a power density of 14 739.4 W kg^−1^, which is more than 14 times higher than that of the bare K (Figure 5e). To further evaluate the compatibility of MoC/NC@K with other electrolyte systems, symmetric cells were tested in an ether‐based electrolyte consisting of 3 m KFSI dissolved in DME. At a current density of 1 mA cm^−2^ and a capacity of 1 mAh cm^−2^, MoC/NC@K exhibits a stable cycling performance for over 2000 h, maintaining a polarization voltage consistently below 55 mV (Figure S19). These results not only highlight the exceptional electrochemical stability of MoC/NC@K but also underscore the broad applicability and robustness of its interfacial architecture across diverse electrolyte environments.

## 3 Conclusions

In conclusion, DFT calculations and experimental analysis reveal that the strong interaction between the highly polar MoC/NC intermediate medium and KFSI induces local charge redistribution, enriching K^+^ near the electrode surface and promoting the formation of anion‐dominated weakly solvated structures. A higher proportion of CIPs and AGGs facilitates the construction of an inorganic‐rich SEI layer dominated by KF, which improves the resist dendrites ability of the SEI. The strong K^+^ affinity and low diffusion energy barrier of MoC/NC further enable rapid ion transport and uniform deposition. As a result, symmetric cells maintain stable cycling for over 800 h at 0.5 mA cm^−2^, while full cells with a PTCDA cathode retain 89.1% of their capacity after 600 cycles at 10  C. The interfacial engineering strategy provides an effective approach for simultaneously regulating solvation structures and SEI composition, offering new insights into anion‐derived SEI design and advancing the development of high‐energy‐density PMBs.

## Funding

This work was supported by the National Natural Science Foundation of China (Grant No.52572208), the China Postdoctoral Science Foundation (Grant No. 2025M780028), the Fundamental Research Funds for the Central Universities (Grant No. N25XQD051), Science and Technology Project of China Hua‐Neng Group (No.HNKJ25‐H76).

## Conflicts of Interest

None of the authors have a conflict of interest to disclose.

## Supporting information




**Supporting File**: advs75327‐sup‐0001‐SuppMat.docx.

## Data Availability

The data that support the findings of this study are available from the corresponding author upon reasonable request.
