# Supplementary material for: Spatially Selective Solvation Chemistry by Local Charge Enrichment for Stable Potassium‐Metal Anodes
Source: Adv Sci (Weinh). 2026 Apr 28;13(38):e75327. doi: 10.1002/advs.75327 (PMC13335597; doi:10.1002/advs.75327)
Supplement: Supplementary file 1 — Supporting File: advs75327‐sup‐0001‐SuppMat.docx. [file ADVS-13-e75327-s001.docx]

**Supporting Information**

**Spatially Selective Solvation Chemistry by Local Charge Enrichment for Stable Potassium-Metal Anodes**

*Lu-Kang Zhao*^†^*, Xi-Ran Zhao*^†^*, Zhimin Ding*^†^**, Yu-Hua Bian, Xuan-Chen Wang, Dongdong Zhao, Yizhuo Zhao, Xuan-Wen Gao*, Zhaomeng Liu and Wen-Bin Luo**

Dr. Z. Ding

School of Metallurgy and Materials Engineering, Liaoning Institute of Science and Technology, Benxi, Liaoning 117004,China

E-mail: [dingzhimin@lnist.edu.cn](mailto:dingzhimin@lnist.edu.cn)

Dr. L. K. Zhao, X. R. Zhao, Y. H. Bian, X. C. Wang, D. Zhao, Y. Zhao, Dr. A/Prof. X. W. Gao, Dr. Z. Liu, Prof. Dr. W. B. Luo

Institute for Energy Electrochemistry and Urban Mines Metallurgy, School of Metallurgy, Northeastern University, Shenyang, Liaoning 110819, China.

E-mail: [luowenbin@smm.neu.edu.cn](mailto:luowenbin@smm.neu.edu.cn)

^†^ These authors contributed equally to this work.

**Experimental Section**

**Synthesis of** **MoC/NC and MoC/NC@K.** The synthesis involves the preparation of C_3_N_4_ and its subsequent ball milling with a molybdenum source. In detail, urea was thermally treated in a muffle furnace at 550 °C for 6 hours. Following the thermal condensation of urea, C_3_N_4_ was successfully synthesized. C_3_N_4_ and ammonium molybdate tetrahydrate ((NH_4_)_6_Mo_7_O_24_·4H_2_O) were mixed at a mass ratio of 2:1 and subsequently ball-milled at approximately 400 rpm for 3 hours. The resulting mixture was then annealed under an argon atmosphere at 800 °C for 2 hours, hereby producing MoC/NC.

The as obtained MoC/NC powder was sprinkled onto metallic potassium and subjected to repeated cold rolling and folding until the surface became uniformly black, yielding the integrated MoC/NC@K electrode. The resulting composite was then punched into 10 mm circular discs for use in symmetric cells, full cells, and other electrochemical measurements. For comparison, pristine potassium metal without modification was used as the control electrode (bare K).

**Characterization Methods.** The phase structure of MoC/NC was analyzed by X-ray diffraction (XRD, EVASTAR-XRD) with a scanning rate of 5° min^-1^. The morphology of the electrodes after cycling was characterized by scanning electron microscopy (SEM, JEOL JSM-7610FPlus), and the elemental distribution was analyzed using energy-dispersive spectroscopy (EDS, Oxford ULTIM MAX 40). X-ray photoelectron spectroscopy (XPS, PHI VersaProbe 4, ULVAC-PHI) was employed to investigate the chemical states of MoC/NC powders and cycled electrodes. Raman spectroscopy (LabRAM HR800, Horiba Jobin Yvon) was used to characterize the structure of MoC/NC powders and the solvation structure of the electrolyte.

**Electrochemical Measurements.** All electrochemical tests were carried out using CR2032 coin cells. Whatman GF/B glass fiber were used as separators, and 140 μL of 1 M KFSI in EC/DEC was used as the electrolyte. PTCDA was employed as the cathode for full-cells. The cathode was prepared by mixing PTCDA, Super P, and PVDF at a mass ratio of 7:2:1, coating onto an aluminum foil current collector, and drying under vacuum at 80 °C. The electrochemical performance of the full cells was evaluated based on the mass of PTCDA, with a PTCDA loading of approximately 3.2 mg cm^-2^. In addition, the energy density and power density were calculated by averaging the specific discharge energy and discharge time from five discharge measurements of PTCDA at each current density. Electrochemical performance was evaluated using a Neware battery testing system (CT-4008Tn-5V20mA-164). Variable-temperature electrochemical impedance spectroscopy (EIS) and in-situ EIS measurements during continuous deposition were performed using the DH7006 electrochemical workstation (Jiangsu Donghua Analytical Instruments Co., Ltd.).

**Theoretical Calculation Methods.** The Materials Studio was used to construct those theoretical models. The CASTEP package^[1]^ in Materials Studio was used to optimize the initial structures. During the structure optimization, ion-electron interactions were determined using the projector-augmented plan wave method, and the Perdew-Burke-Ernzerhof (PBE) exchange-correlation functions were used to calculate the generalized gradient approximation (GGA).^[2-4]^ The convergence criteria for energy and force were set at 2×10^-6^ eV/atom and 0.1 eV/Å, respectively, and the plane-wave energy cutoff was 600 eV. A gamma-centered 1×1×1 k-point grid was adopted for sampling. A vacuum layer of 30 Å was constructed over the surface normally to avoid interaction between periodic images and facilitate molecular adsorption. After initial structure optimization, using the Vienna Ab-initio Simulation Package (VASP) to optimize the structure further and calculate the adsorption energy of K^+^. The Perdew–Burke–Ernzerhof (PBE) functional within the generalized gradient approximation (GGA) was also used in the static calculation process. A Gaussian smearing method with a width of 0.05 eV was applied. The convergence criteria for energy and force were set at 10^-6^ eV/atom and 0.02 eV/Å, respectively, and the plane-wave energy cutoff was 520 eV. A gamma-centered 1×1×1 k-point grid was adopted for sampling. To balance the charge when modeling K^+^ adsorption, an FSI^-^ anion was placed in the vacuum layer randomly.

Van der Waals interactions were corrected using the Grimme DFT-D3 method.^[5]^ The adsorption energy (E_ads_) was calculated as the following equation:

*E*_ads_ *= E*_slab+mol_ *– E*_slab_ *− E*_mol_ (1)

where the E_slab+mol_, the E_slab_, and the E_mol_ represent the total energy of adsorbates on the material surface, the energy of the material surface, and the energy of the isolated adsorbates, respectively.

The differential charge density was calculated as the following equation:

$\text{Δ}\text{ρ=}\text{ρ}_{\text{total}}\text{-}\text{∑}\text{ }\text{ρ}_{\text{i}}$ (2)

where $\text{Δ}\text{ρ}$ represents the differential charge density, $\text{ρ}_{\text{total}}$ is the charge density of the complete system, and $\text{ρ}_{\text{i}}$ is the sum of the charge densities of individual components.

where $\text{Δ}\text{ρ}$ represents the differential charge density, $\text{ρ}_{\text{total}}$ is the charge density of the complete system, and $\text{ρ}_{\text{i}}$ is the sum of the charge densities of individual components.

The migration barrier for K^+^ ions was calculated using the climbing image nudged elastic band (CI-NEB) method.^[6]^

Additionally, the nucleophilic reactivity of solute molecules was analyzed using DFT combined with the Fukui function, computed by the DMol3^[7]^ module in Materials Studio. The convergence threshold for the electronic energy was set to 0.02 eV. The nucleophilic Fukui function was calculated using:

$\text{ f}^{\text{+}}\text{=}\text{q}\left( \text{N}\text{+1} \right)\text{-}\text{q(N)}$ (3)

where $\text{f}^{\text{+}}$is the nucleophilic property, $\text{q}\left( \text{N+1} \right)$ is the electron density after adding one electron, and $\text{q}\left( \text{N} \right)$ is the electron density of the neutral system, respectively.

**COMSOL Simulation.** In a simplified two-dimensional model, finite element analysis was conducted by coupling the tertiary current distribution with the level set physics module. The simulation domain was set to 25 × 20 μm. The fluid dynamics mesh utilized a extra refine free triangular mesh, and add refinement near the electrode boundaries. The model compared the deposition growth behavior of bare K versus K with MoC /NC protective layer.


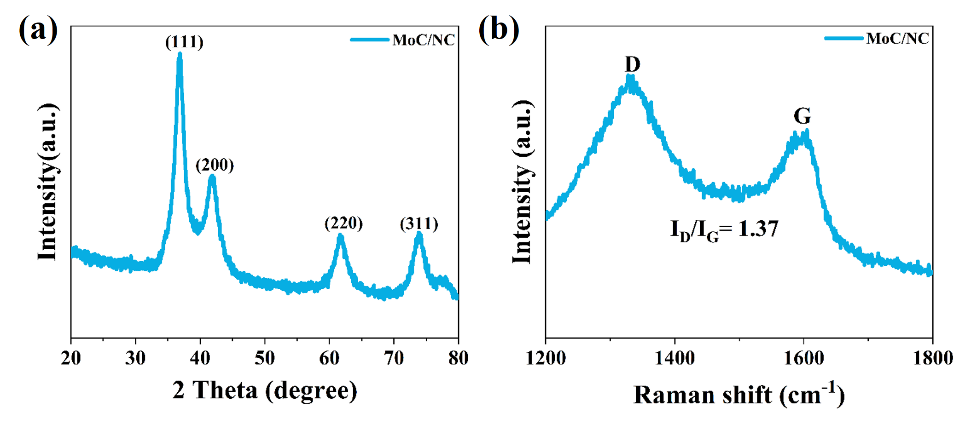


**Figure S1.** (a) XRD patterns and (b) Raman spectra of MoC/NC.


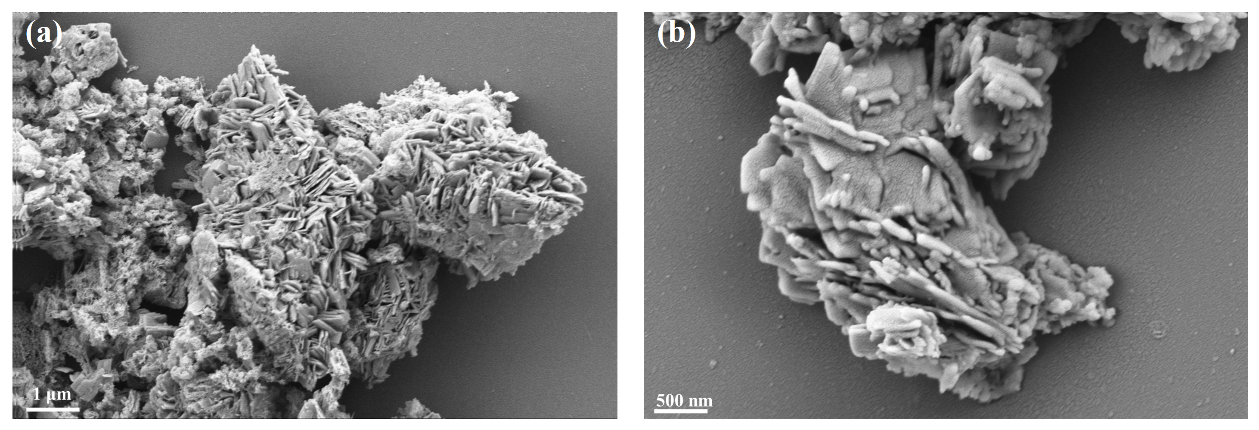


**Figure S2.** SEM images of the MoC/NC.


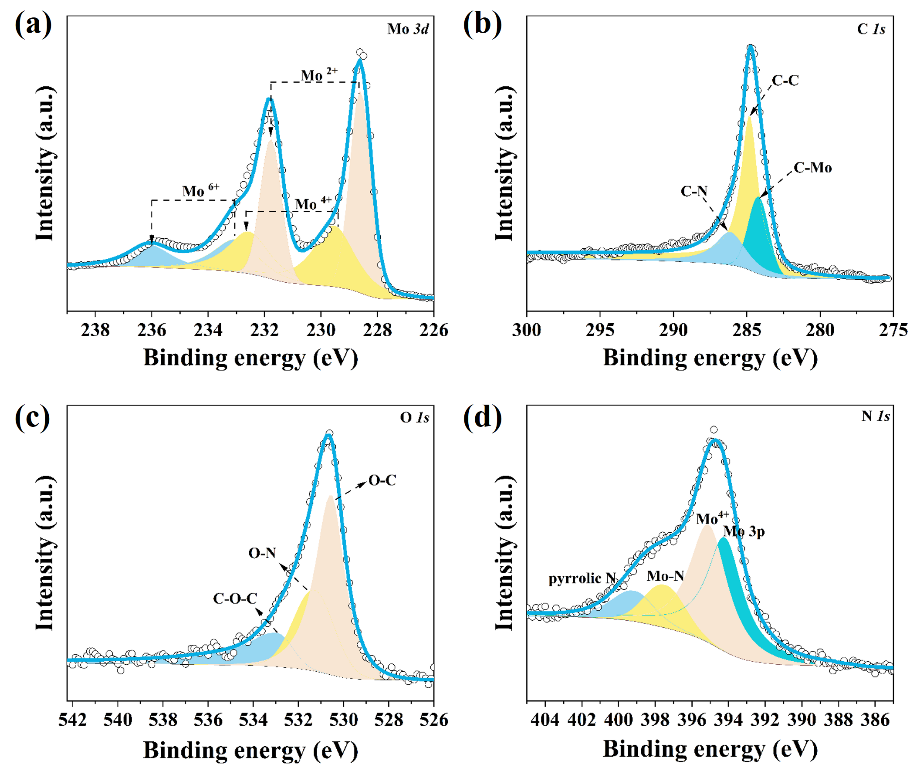


**Figure S3.** High-resolution XPS spectra of the MoC/NC. (a) Mo *3d*, (b) C *1s*, (c) O *1s* and (d) N *1s.*


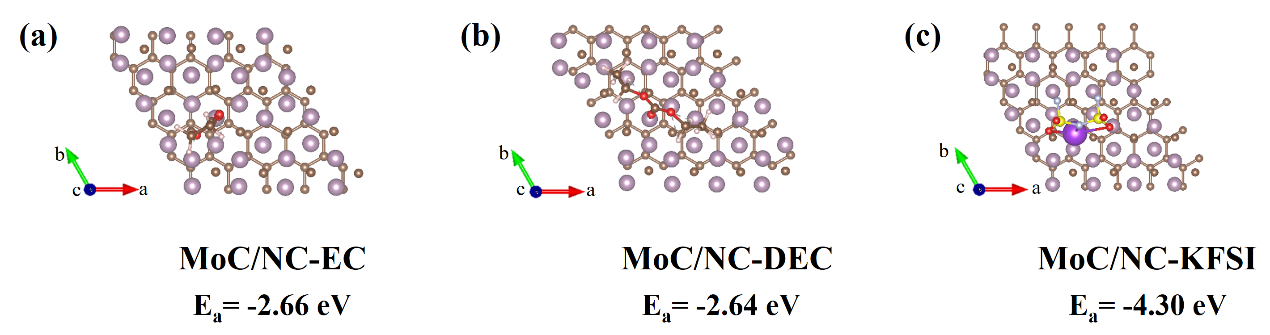


**Figure S4.** Adsorption energy of (a) EC, (b) DEC and (c) KFSI with the MoC/NC.

*
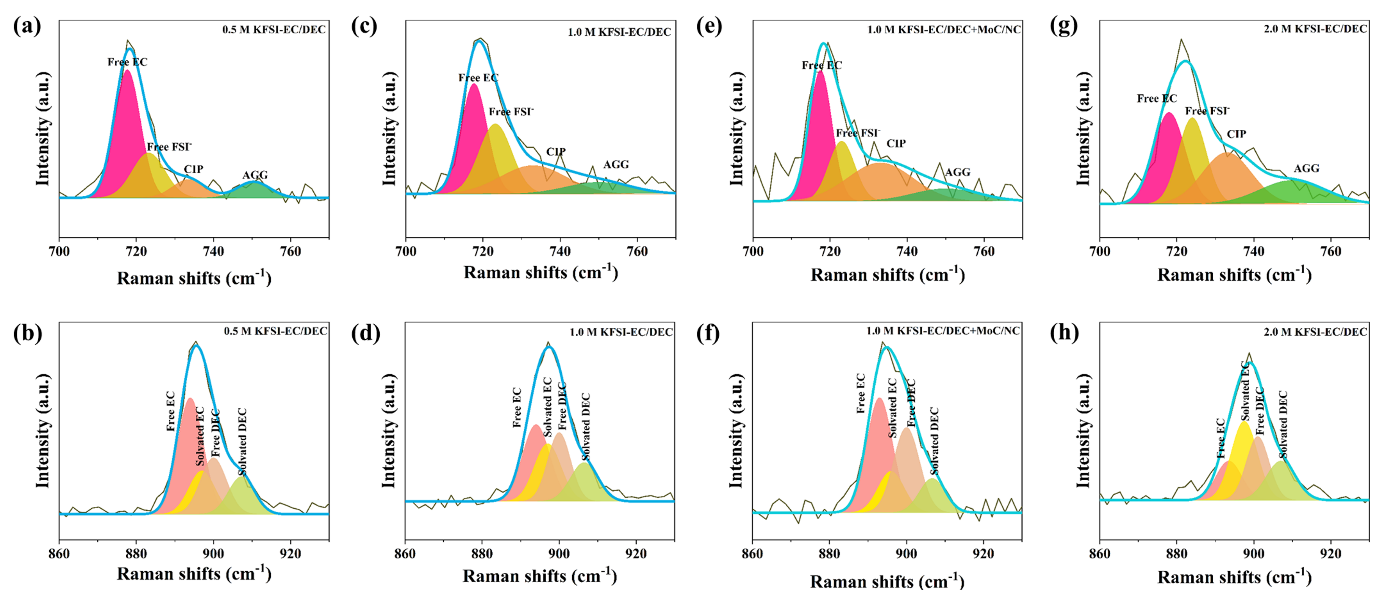
*

**Figure S5.** Raman spectra in the electrolyte of (a-b) 0.5 M KFSI-EC/DEC, (c-d) 1.0 M KFSI-EC/DEC, (e-f) 1.0 M KFSI-EC/DEC with MoC/NC, and (g-h) 2.0 M KFSI-EC/DEC in the range of 700-770 cm^-1^ and 860-930 cm^-1^, respectively.

*
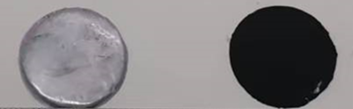
*

**Figure S6.** The digital photograph of the bare K (left) and MoC/NC after compounding with metallic K (right).


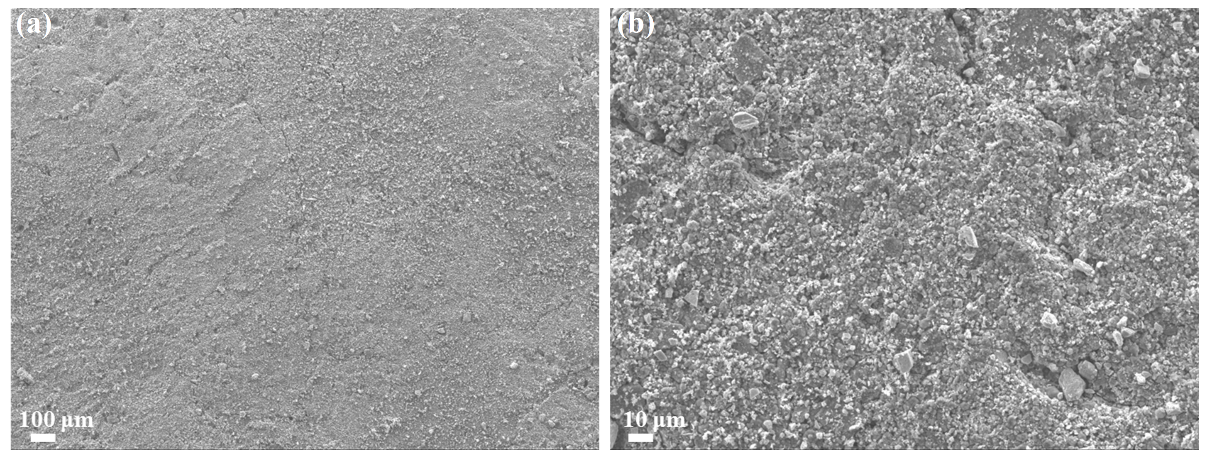


**Figure S7.** The SEM images of MoC/NC after compounding with metallic K.


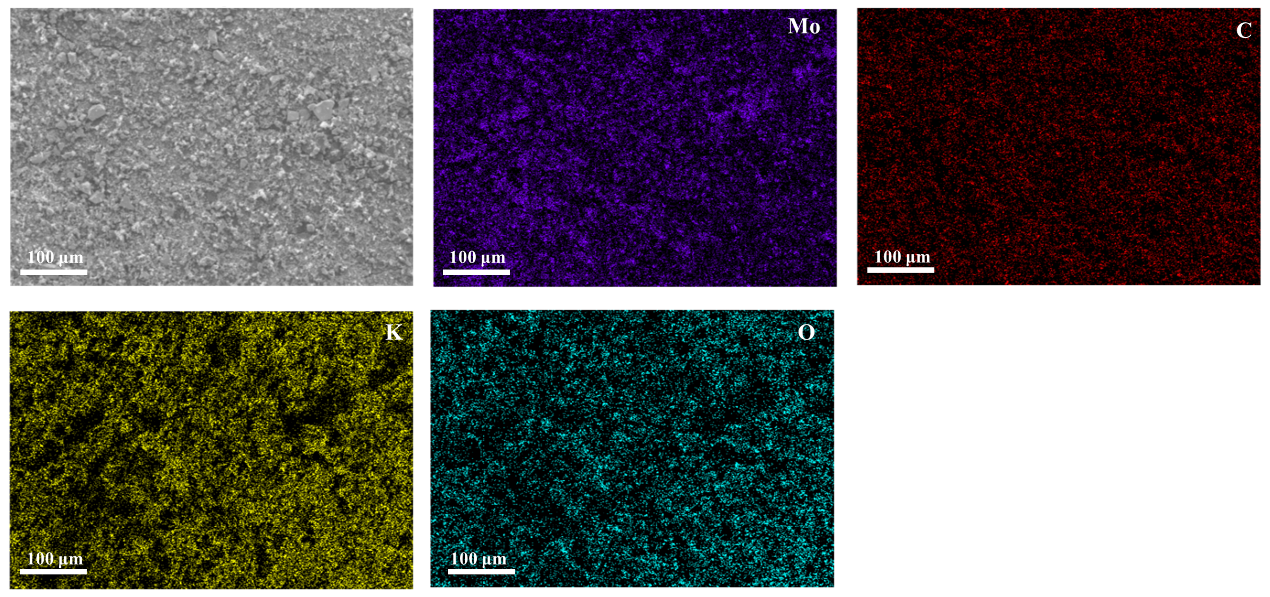


**Figure S8.** The EDS element distribution mapping of MoC/NC after compounding with metallic K.


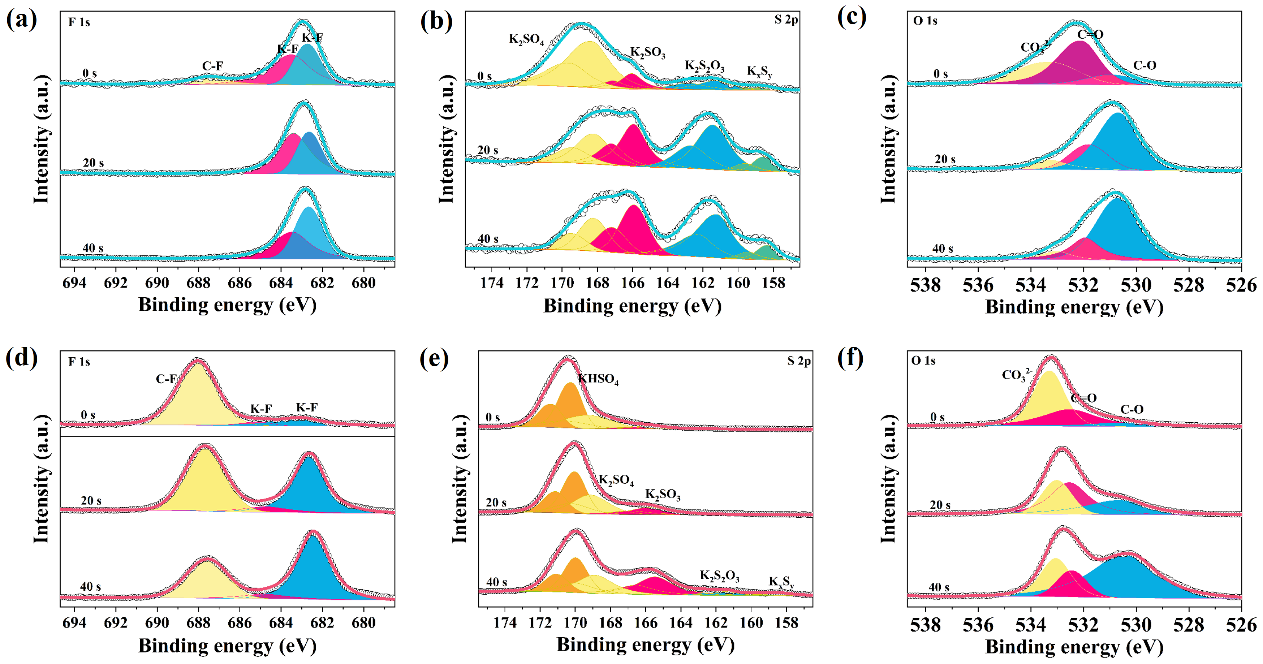


**Figure S9.** XPS spectra of the MoC/NC@K and bare K after cycling. (a) F *1s*, (b) S *2p* and (c) O *1s* high-resolution XPS spectra of the MoC/NC@K. (d) F *1s*, (e) S *2p* and (f) O *1s* high-resolution XPS spectra of the bare K.


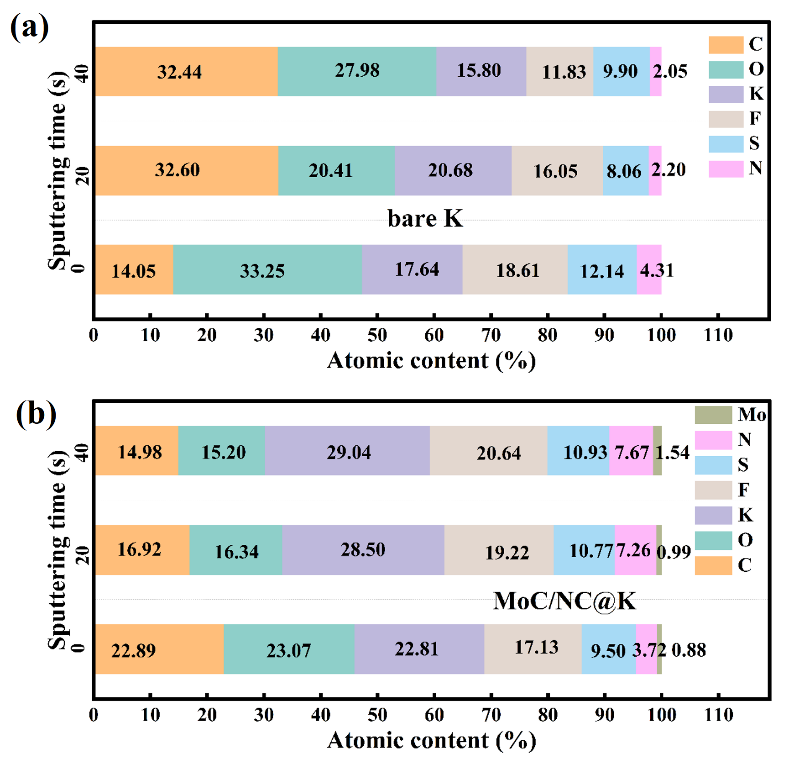


**Figure S10.** Analysis of surface chemical elements of (a) bare K and (b) MoC/NC@K after cycling.


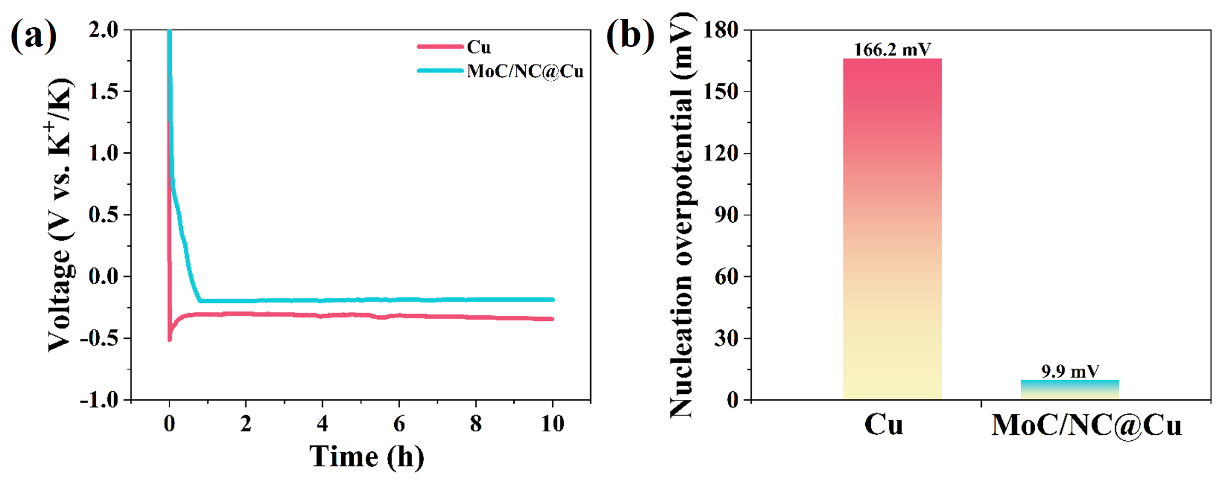


**Figure S11.** The nucleation overpotential of K on Cu and MoC/NC@Cu.


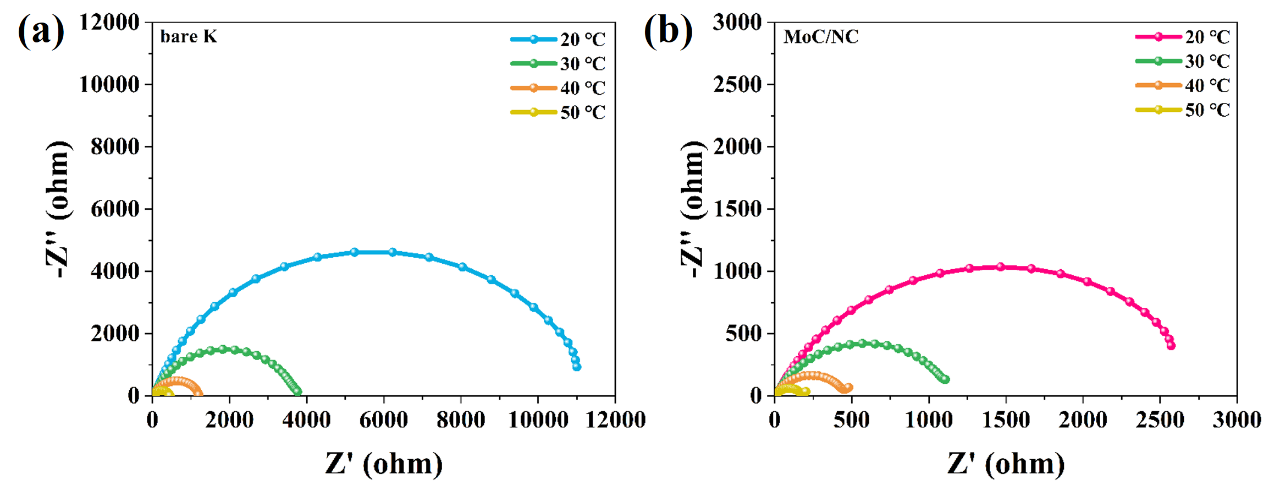


**Figure S12.** The temperature-variable impedance of (a) bare K and (b) MoC/NC@K.


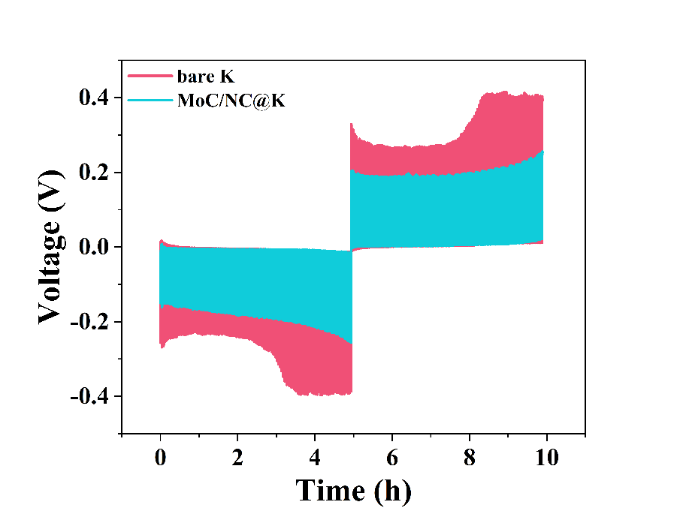


**Figure S13.** The GITT curves of bare K and MoC/NC@K.


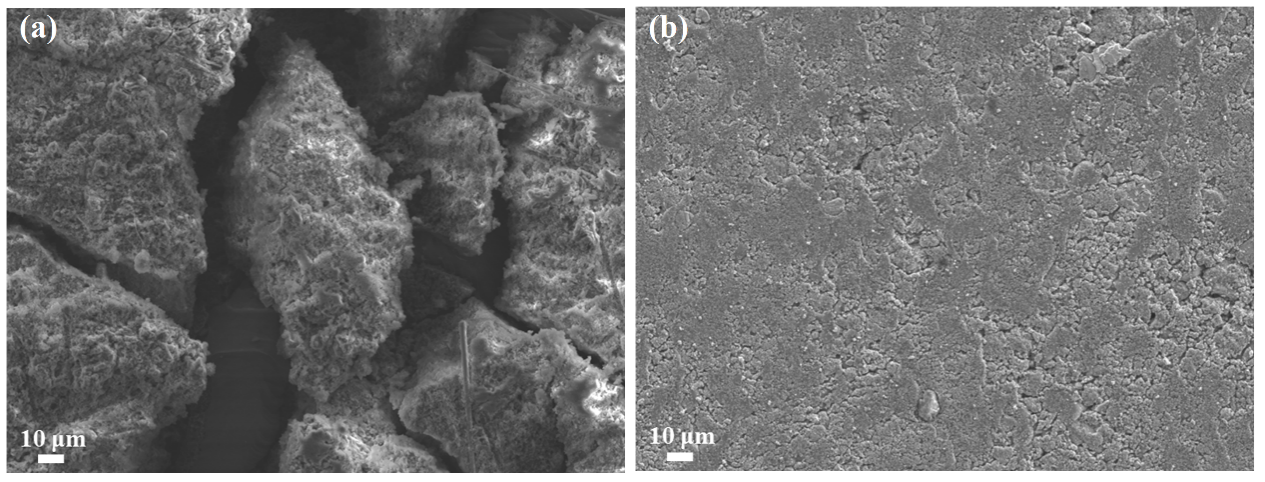


**Figure S14.** SEM images of the (e) bare K and (f) MoC/NC@K after long-term cycling.


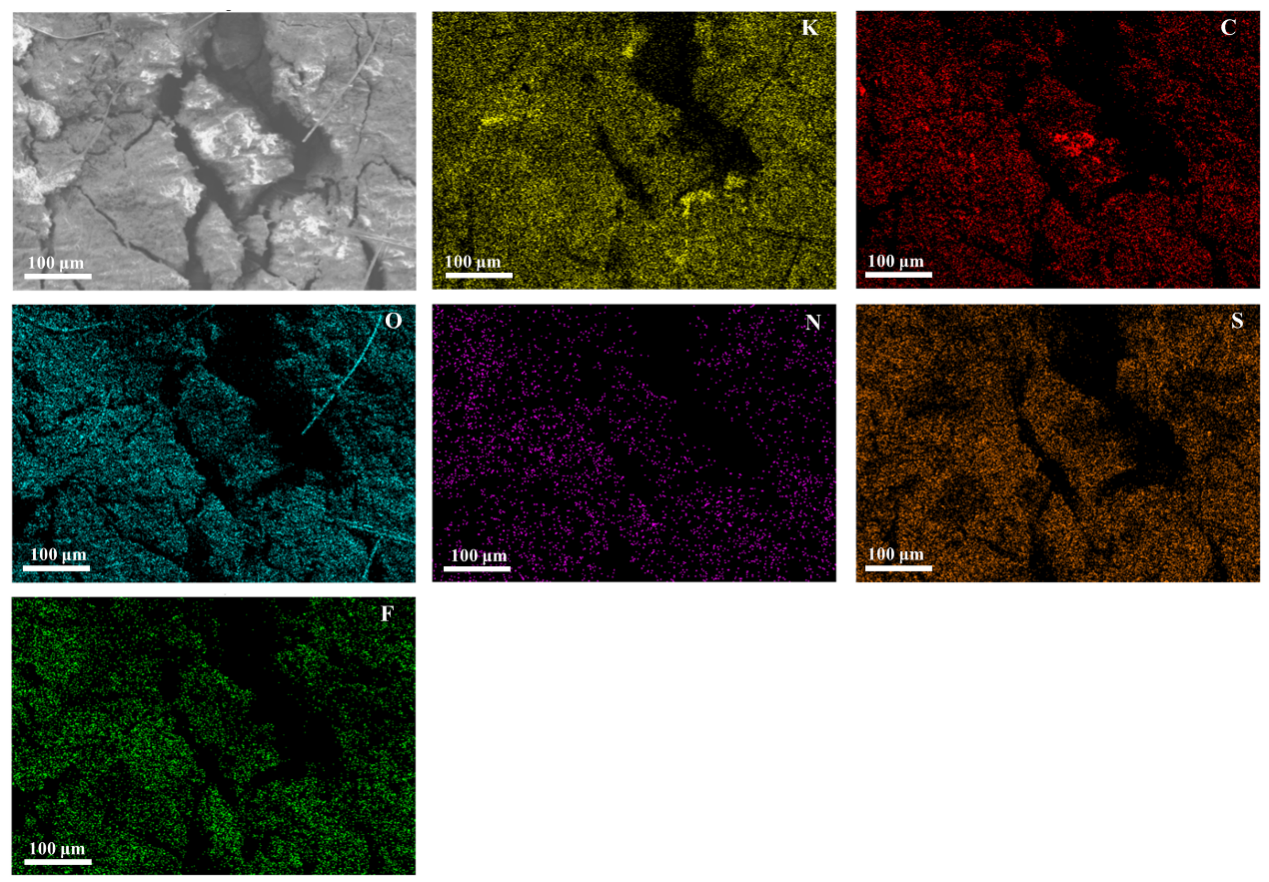


**Figure S15.** EDS elemental distribution mapping of the bare K after cycling.


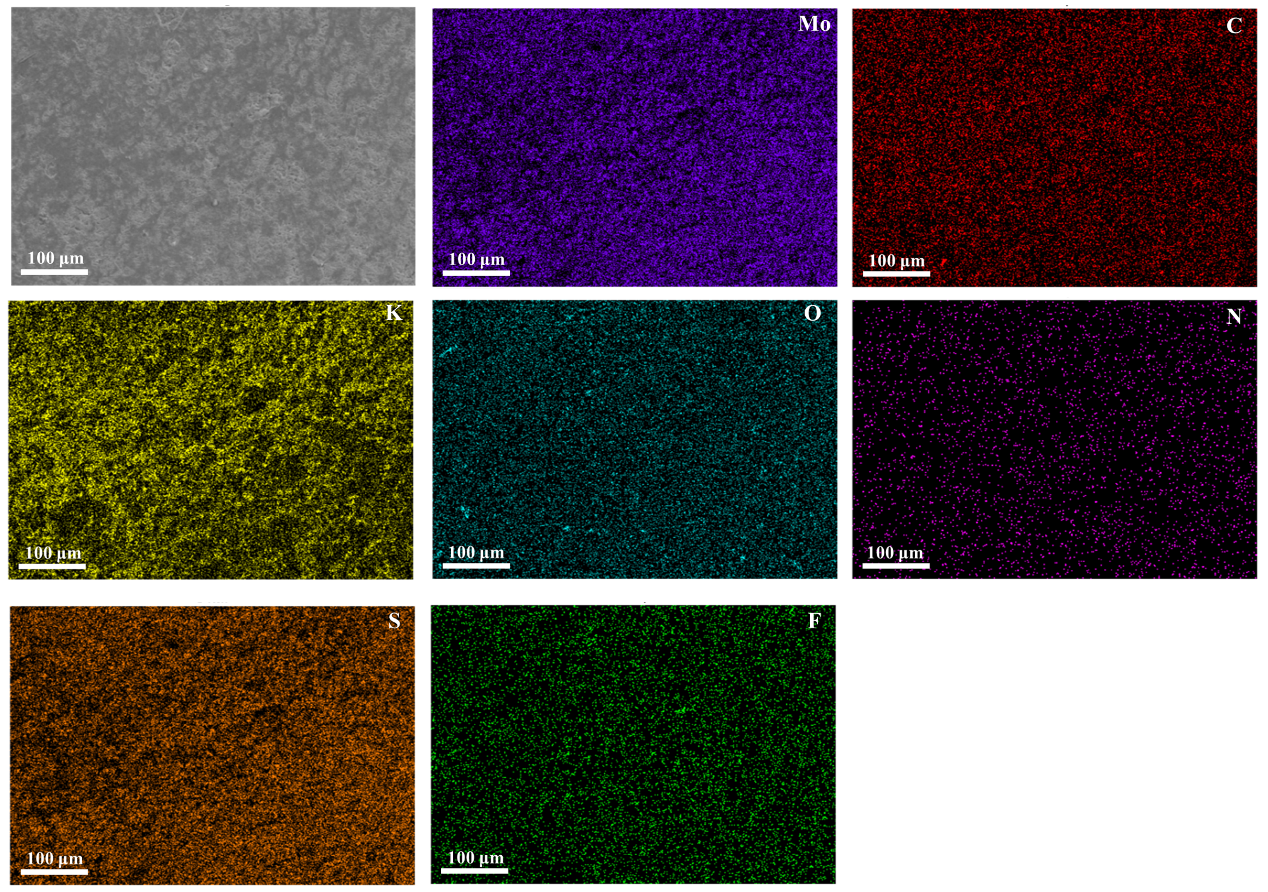


**Figure S16.** EDS elemental distribution mapping of the MoC/NC@K after cycling.


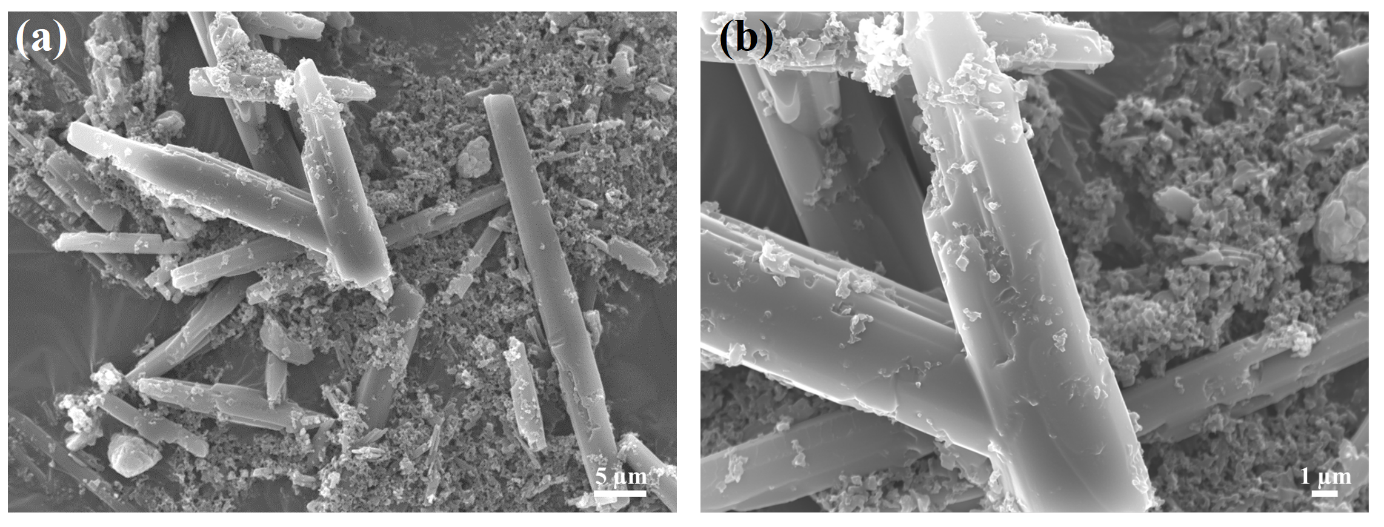


**Figure S17.** SEM images of PTCDA after annealing at 450 ℃ for 4 hours.

*
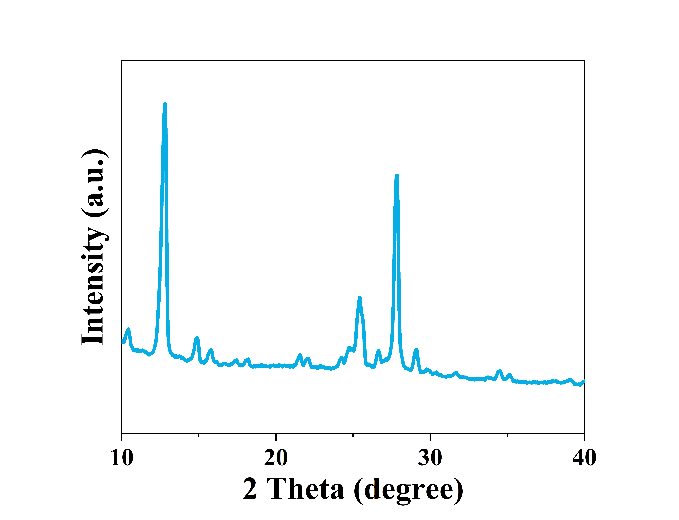
*

**Figure S18.** XRD pattern of PTCDA after annealing at 450 ℃ for 4 hours.


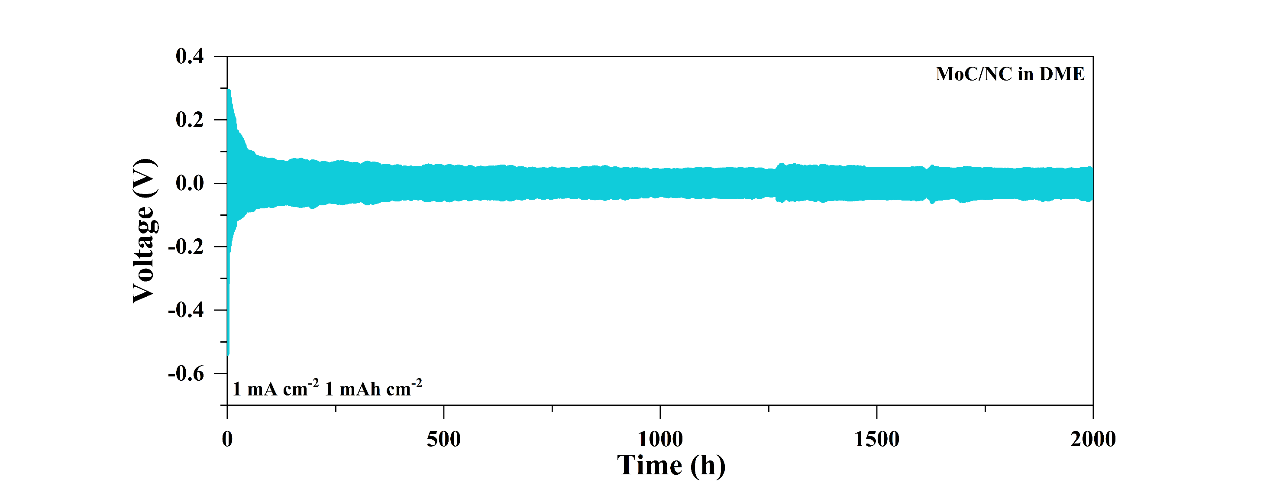


**Figure S19.** The repeated deposition/stripping performance of the MoC/NC@K electrode in the 3.0 M KFSI-DME electrolyte.

**Table S1**. The calculated Fukui functions value of KFSI and FSI^-^ anions

| KFSI | | | | FSI^-^ | | |
| --- | --- | --- | --- | --- | --- | --- |
| Atom | f^+^ | f^0^ | f^+-^ f^0^ | f^+^ | f^0^ | f^+-^ f^0^ |
| S | 0.019 | 0.045 | -0.026 | 0.066 | 0.067 | -0.001 |
| O | 0.047 | 0.094 | -0.047 | 0.144 | 0.15 | -0.006 |
| O | 0.018 | 0.071 | -0.053 | 0.126 | 0.136 | -0.01 |
| F | 0.011 | 0.034 | -0.023 | 0.06 | 0.06 | 0 |
| N | -0.003 | 0.075 | -0.078 | 0.207 | 0.174 | 0.033 |
| S | 0.019 | 0.045 | -0.026 | 0.066 | 0.067 | -0.001 |
| O | 0.047 | 0.094 | -0.047 | 0.144 | 0.15 | -0.006 |
| O | 0.018 | 0.071 | -0.053 | 0.126 | 0.136 | -0.01 |
| F | 0.011 | 0.034 | -0.023 | 0.06 | 0.06 | 0 |

**Table S2**. Peak area percentages of the four electrolytes obtained by fitting in the range of 700-770 cm^-1^.

| Electrolyte | Free EC | Free FSI^-^ | CIP | AGG |
| --- | --- | --- | --- | --- |
| 0.5 M-KFSI-EC/DEC | 56.282 % | 24.701 % | 10.147 % | 8.870 % |
| 1.0 M-KFSI-EC/DEC | 36.997 % | 29.310 % | 23.812 % | 9.881 % |
| 1.0 M-KFSI-EC/DEC+MoC/NC | 37.876 % | 19.868 % | 31.786 % | 10.470 % |
| 2.0 M-KFSI-EC/DEC | 30.843 % | 25.686 % | 26.946 % | 16.525 % |

**Table S3**. Peak area percentages of the four electrolytes obtained by fitting in the range of 860-930 cm^-1^.

| Electrolyte | Free EC | K^+^-EC | Free DEC | K^+^-DEC |
| --- | --- | --- | --- | --- |
| 0.5 M-KFSI-EC/DEC | 45.857 % | 17.142 % | 22.199 % | 14.802 % |
| 1.0 M-KFSI-EC/DEC | 33.289 % | 24.575 % | 24.987 % | 17.149 % |
| 1.0 M-KFSI-EC/DEC+MoC/NC | 41.704 % | 15.060 % | 30.911 % | 12.325 % |
| 2.0 M-KFSI-EC/DEC | 17.971 % | 36.244 % | 25.536 % | 20.249 % |

**References**

1. Y. Xu, *Powder Technol.* 2021, **377**: 843–846

2 Blöchl, P. E., *Phys. Rev. B* 1994**,** ***50***, 17953.

3. Kresse, G.; Furthmüller, J., *Phys. Rev. B* 1996**,** ***54***, 11169.

4. Perdew, J. P.; Burke, K.; Ernzerhof, M., *Phys.l rev. lett.* 1996, ***77***, 3865.

5. Caldeweyher, E.; Bannwarth, C.; Grimme, S., *J. chem. Phys.* 2017, ***147****, 034112*.

6. G. Henkelman, B. P. Uberuaga, H. Jonsson, *J. Chem. Phys.* 2000, **113**, 9901.

7. Delley B.; *Comput. Mat. Sci.* 2000, **17**(2-4): 122-126.
